# Gas Sensing with Bare and Graphene-covered Optical Nano-Antenna Structures

**DOI:** 10.1038/srep21287

**Published:** 2016-02-17

**Authors:** Bhaven Mehta, Kurt D. Benkstein, Steve Semancik, Mona E. Zaghloul

**Affiliations:** 1 Department of Electrical and Computer Engineering, The George Washington University, Washington DC, 20037; 2 Biomolecular Measurement Division, National Institute of Standards and Technology, Gaithersburg, MD 20899-8362.

## Abstract

The motivation behind this work is to study the gas phase chemical sensing characteristics of optical (plasmonic) nano-antennas (ONA) and graphene/graphene oxide-covered versions of these structures. ONA are devices that have their resonating frequency in the visible range. The basic principle governing the detection mechanism for ONA is refractive index sensing. The change in the concentration of the analyte results in a differing amount of adsorbate and correlated shifts in the resonance wavelength of the device. In this work, bare and graphene or graphene oxide covered ONA have been evaluated for gas sensing performance. Four different analytes (ethanol, acetone, nitrogen dioxide and toluene) were used in testing. ONA response behavior to different analytes was modified by adsorption within the graphene and graphene oxide overlayers. This work is a preliminary study to understand resonance wavelength shift caused by different analytes. Results imply that the combination of well-structured ONA functionalized by graphene-based adsorbers can give sensitive and selective sensors but baseline drift effects identified in this work must be addressed for applied measurements.

Presently, solid-state sensors are most prominently used for gas phase chemical detection^1^. In these sensors, there is a change in a physical property such as the conductance^1^,^2^ or the resonance frequency^3^ because of the interaction of the gases with the sensing material. Such physical property changes are then transduced into an electrical signal, which is measured. Researchers employ sensing materials that are sensitive to the gas, which can be a 1-D material like nanowires^4^ and nanotubes^5^, or it can be a 2-D material like graphene^1^, or bulk type materials. However, there can be disadvantages associated with sensors based on 1-D materials, such as the noise and resistance introduced by the contacts^6^, operation at elevated temperature for better selectivity^7^, and the use of dielectrophoresis for alignment in case of 1-D materials^4^.

Plasmonic structures have been widely used for biological sensing^8^,^9^. Shifts in the resonance frequency are measured as a result of changes in the concentration of the adsorbed analyte molecules. Plasmonics structures have been rarely used for gas phase chemical sensing. This can be attributed to the relatively small changes observed in the resonance frequency for different concentration of gases^10^,^11^ resulting from the small size of the analyte gas molecules as compared to the size of biological molecules.

In this work, an array of dipole structures is designed as an ONA with resonance frequency in the visible spectrum. The array structure enhances the spectrum response of the ONA. The ONA was chosen with a bulk sensitivity of 450 nm/RIU. Response of the bare ONA for four different analytes (acetone, ethanol, toluene and nitrogen dioxide) was studied. A shift of 0.5 nm to 3.5 nm in the resonance peak was observed. Motivated by a desire to attain greater sensitivity and selectivity than the bare ONA, we tested the same four analytes on graphene coated and graphene oxide coated ONA structures. We observed a much larger frequency shift with graphene and graphene oxide covered ONA. Bare ONA exhibits only a red shift when exposed to all the analytes. Graphene and graphene oxide coated ONA exhibited a blue shift when exposed to toluene and a red shift when exposed to the other three analytes. In this work, there was no baseline drift observed for the bare ONA sensor. However, graphene and graphene oxide coated ONA exhibited baseline drift apparently arising from residual adsorbed gas molecules. We propose a suggested mechanism for the frequency shifts observed for graphene and graphene oxide coated ONA for different analytes.

## Experimental

### Materials and Methods

**Figure 1:**
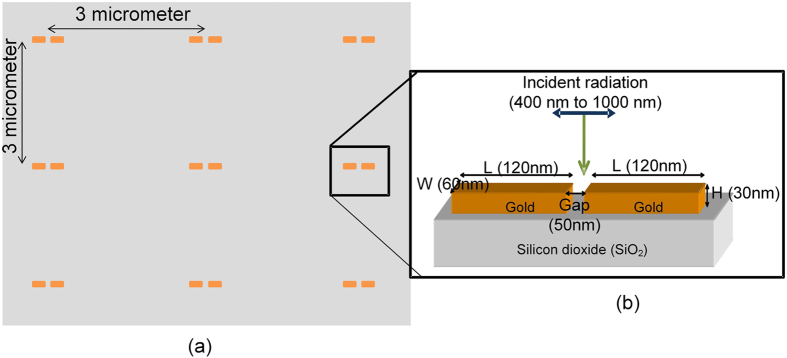
Dipole nano antenna structure made with gold on a fused silica substrate. (**a**) Top view of the array of the ONA. Background (in gray) is fused silica. (**b**) Focused side view of the ONA.

**Figure 2:**
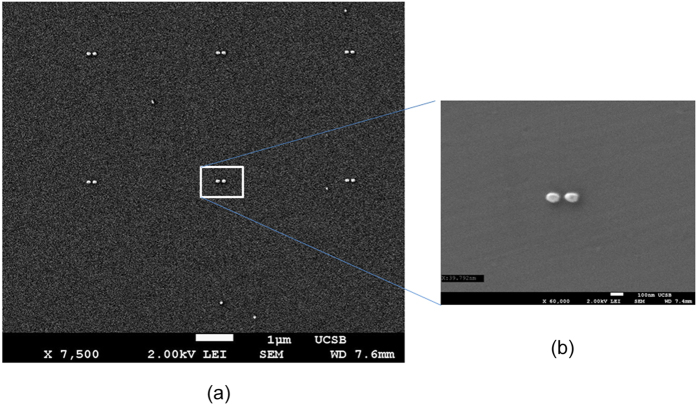
(**a**) SEM images of the fabricated array of ONA array. (**b**) SEM image of an ONA with rounding.

ONA were prepared using e-beam lithography, metal deposition and lift-off as mentioned in the literature^12^. The gap between the two consecutive dipoles was 3 *μ*m as shown in Fig. 1. This gap is three times the highest incident wavelength used (1000 nm) to avoid any interference. The dipole was fabricated in gold with length, width, thickness and gap between the arms of 120 nm, 60 nm, 30 nm and 50 nm, respectively, as shown in Fig. 1b. The comparison between the simulated response and the experimental measured resonance spectrum is shown in the Supporting Information (Figure S1). As seen in Fig. 2, rounding occurs at the corners of dipole sectors during the fabrication process. The effect of rounding is discussed in more detail in Section 1 of the Supplementary information. The rounding of the dipole structures results in a blue shift in the resonance wavelength compared to the expected value. Along with the rounding, the random variations in the fabrication process result in the broadening of the spectral response. Graphene was purchased from CVD Equipment Corporation (Disclaimer) and graphene oxide was purchased from Graphene Supermarket (Disclaimer) for these experiments. Graphene oxide was drop cast onto the sample and dried in ambient room temperature conditions.

### Testing Setup

**Figure 3:**
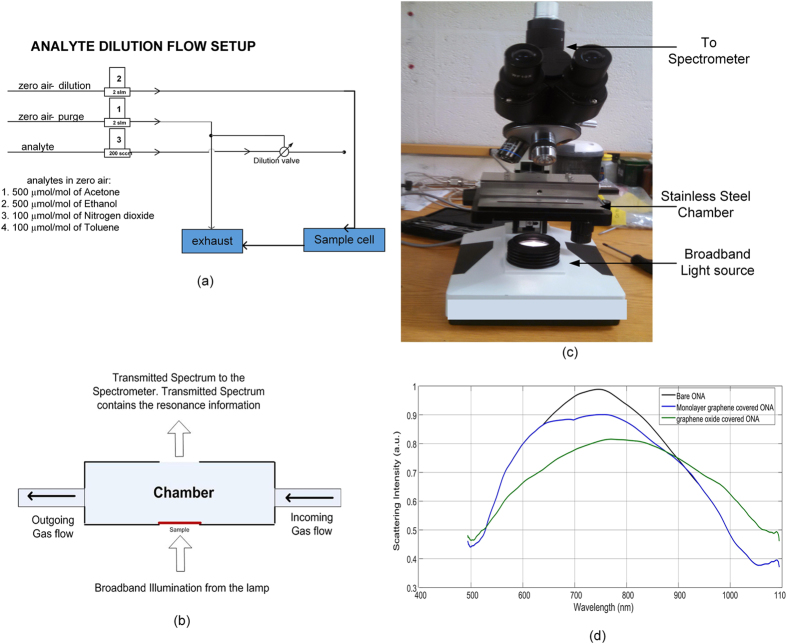
Schematics of (**a**) analyte flow system, and (**b**) transmission setup used. (**c**) Microscope setup with gas chamber. (**d**) Measured spectral response of bare ONA, monolayer graphene covered ONA and graphene oxide covered ONA.

The testing setup is as shown in Fig. 3. The setup can be divided into two parts, namely, the analyte delivering part (as shown in Fig. 3a) and the resonance measurement setup (as shown in Fig. 3c). Figure 3b shows an expanded view of the flow-through chamber that is incorporated into the Fig. 3c setup. The analyte was allowed to flow through the stainless steel chamber on top of the microscope as shown in Fig. 3b. The ONA sample in the chamber was illuminated from below by broadband light as shown in Fig. 3c.

Four analytes (ethanol, acetone, nitrogen dioxide and toluene) were tested on the three different ONA configurations. Three configurations of ONAs were tested: (i) bare ONA, (ii) graphene covered ONA and (iii) graphene oxide covered ONA. Acetone and ethanol with concentrations of (5, 20, 50 and 100) *μ*mol/mol, and toluene and nitrogen dioxide with concentrations of (1, 4, 10 and 20) *μ*mol/mol were examined. Analytes were delivered from cylinders, pre-diluted with zero air, and then further diluted to testing levels using our gas-flow manifold (Fig. 3a). While maintaining a constant flow rate of 1 standard liter/min, the ONA was exposed to the analytes in air using 2 min pulses, alternated with 4 min pulses of the dry-air background. The analyte was allowed to flow through the stainless steel chamber on top of the microscope as shown in Fig. 3b. The chamber had the ONA sample illuminated from the bottom by broadband light as shown in Fig. 3c. Transmitted light was collected using a 10x objective with numerical aperture NA = 0.25 onto the CCS 175 spectrometer (500 nm to 1000 nm) from Thorlabs (Disclaimer). The integration time was set to be 3 seconds. The samples were collected at an interval of 5 seconds. The spectrometer was set to box averaging of 50x.

### Data Analysis

The intensity was determined by the following Equation 1,


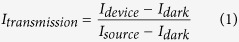


where *I*_*transmission*_ is the transmission spectrum of the device. *I*_*device*_ is the spectrum when ONA are illuminated. *I*_*source*_ is the spectrum of the light passing through the fused silica without the ONA. *I*_*dark*_ is the spectrum collected when the light source is turned off. The data processing was done using MATLAB (Disclaimer). A linear fit was used to determine the slope of concentration-wavelength shift curve for a given device. The linear fit was used only for those readings for which the response was not saturated. We don’t observe any variation in the resonance wavelength beyond the measurement capabilities of the spectrometer (accuracy of 0.6 nm, resolution of 0.15 nm). In these feasibility studies each sample was tested once for all the analytes.

## Results and Discussion

In this work, we study the change in the resonance frequency of ONA when exposed to gas-phase analytes. We note that this proof-of-concept study uses three different ONA arrays to demonstrate the feasibility of using them for gas-phase sensing. Furthermore, we explored the possible utility of employing modifying layers to affect the response phenomena for two of the ONA arrays. The shift in the resonance wavelength, Δ*λ*, is given by Equation 2,^8^.


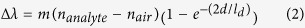


where, *n*_*analyte*_ and *n*_*air*_ are the refractive indices of the analyte and air. *d* is the thickness of the analyte layer on top of the dipole structure. *l*_*d*_ is the exponential field decay length. *m* is the bulk refractive index sensitivity of the bare ONA. For graphene/graphene oxide covered ONA, *m* is the bulk refractive index sensitivity of the combined device (ONA + graphene or graphene oxide). Using Lumerical’s FDTD (Finite Difference Time Domain) tool (Disclaimer) we determined that *m* is similar for bare ONA and graphene/graphene oxide covered ONA (which lies in the range of 450 nm/RIU to 730 nm/RIU). The values in Table 1 allow one to predict expected shifts measured for different conditions. From FDTD simulations^13^, we can also say that rounded corners of the dipole (rounding radius varying from 5 nm to 30 nm) increase the sensitivity of the device.

### Bare ONA

**Figure 4:**
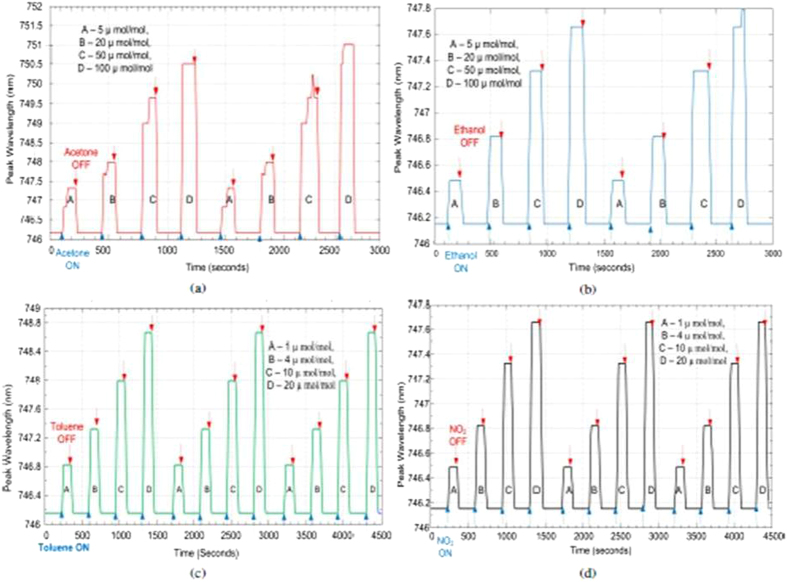
Bare ONA response to (**a**) acetone, (**b**) ethanol, (**c**) toluene and (**d**) nitrogen dioxide.

Based on Equation 2, a red shift (Δ*λ* > 0) is predicted for all the analytes used. The responses of the bare ONA are shown in Fig. 4. There is, in fact, a red shift in the resonance frequency for the four analytes that increases with analyte concentration. The spikes observed for the acetone and ethanol responses of the bare ONA are attributed to random vibrations during the measurement cycle. The change in the wavelength due to the spike is very small and is of the same magnitude as the resolution of the spectrometer. Note that the responses of the bare ONA return fully to their baseline value when the analyte flow is stopped, indicating that the detection process is reversible. The shift in the resonance frequency is saturated at higher concentrations (50 *μ*mol/mol and 100 *μ*mol/mol for acetone and ethanol, and 10 *μ*mol/mol and 20 *μ*mol/mol for nitrogen dioxide and toluene). Bare ONA also has a fast response to different analytes. Bare ONA exhibits the highest sensitivity to toluene, which can be attributed to the high refractive index of toluene amongst all four analytes.

### Modified ONA

Motivated by a desire to attain greater sensitivity and selectivity, we also tested two more sensor types with the same four analytes. We characterized the modifying layers with Raman spectroscopy to estimate the coverage level. From the Raman spectra of the covered ONA, we estimate that there are more than 4 layers of graphene oxide stacked on top of each other, while we observe evidence for only a monolayer of graphene covering the ONA (See Figure S4 and S5 in Section 2 of the Supporting Information).

**Figure 5:**
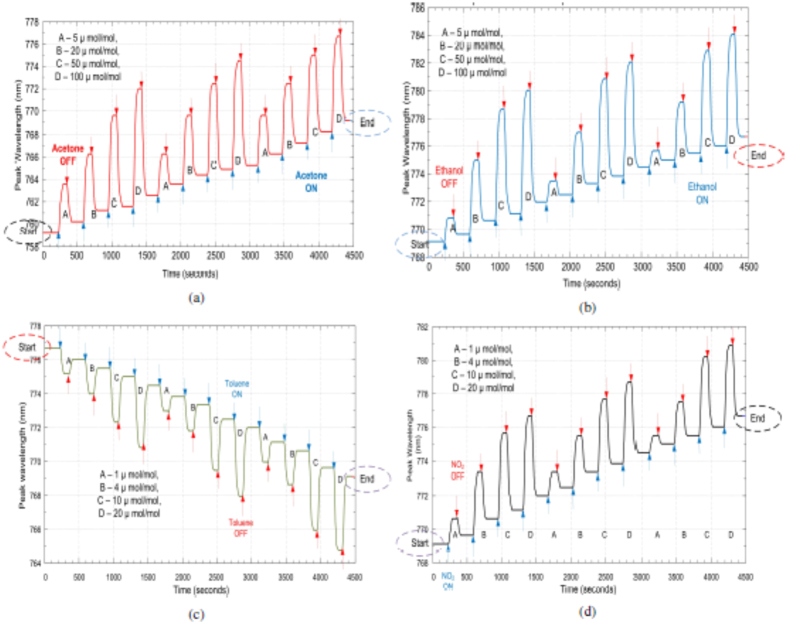
Graphene covered ONA response to (**a**) acetone, (**b**) ethanol, (**c**) toluene and (**d**) nitrogen dioxide.

The response of graphene covered ONA for four different analytes is as shown in Fig. 5. From Equation 2, we expected to have a red shift (Δ*λ* > 0) for the graphene-covered ONA, in response to all the analytes, but a blue shift (Δ*λ* < 0) is observed for toluene as shown in Fig. 5c. We also note that the responses of graphene-covered ONA are qualitatively slower than the bare ONA. This implies that it takes time for the analyte molecules to reach the active portions of the ONA, and be removed from the graphene covering. We also observe that the responses of graphene-covered ONA do not return to the baseline values, which implies that the analyte molecules build up on the graphene layer. Accumulation of analyte molecules has also been observed in graphene-based conductance sensors^14^, indicated by the lack of recovery to the original baseline value after the analyte flow is stopped. It is also interesting to observe the history dependent behavior of the response for graphene covered ONA, which is dependent upon the order of the test gases presented. The history dependence involved a drifting baseline which is clearly visible in Fig. 5. The value of the resonance frequency at which the preceding gas ends is the value from where the succeeding gas begins its response, as highlighted by the circled values in Fig. 5.

**Figure 6:**
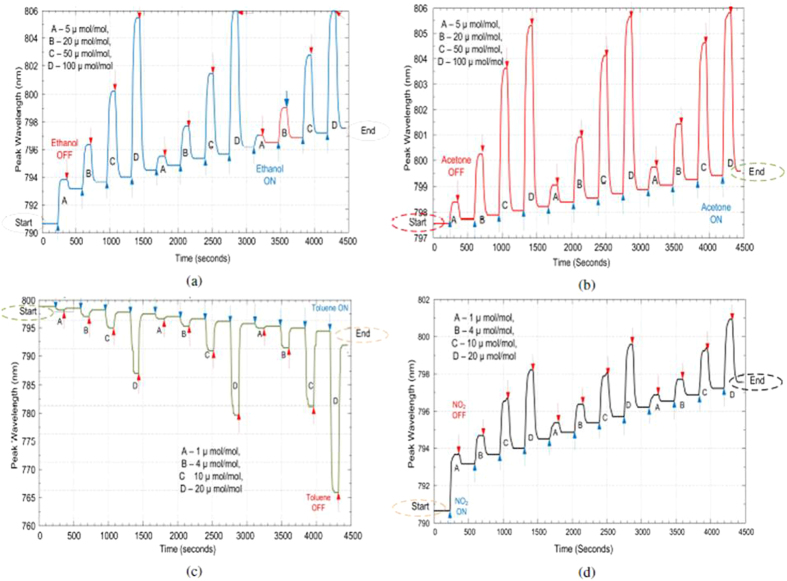
Graphene oxide ONA response to (**a**) ethanol, (**b**) acetone, (**c**) toluene and (**d**) nitrogen dioxide.

The responses of graphene oxide covered ONA are shown in Fig. 6. The responses of the graphene oxide covered ONA are similar to graphene covered ONA. We also observe that responses of graphene oxide-covered ONA do not return to the pre-exposure baselines. The responses of graphene oxide covered ONA are again larger and slower than the bare ONA. We also monitored the dependence of the graphene oxide covered ONA response on the order of gases tested (ethanol, acetone, toluene and nitrogen dioxide). We note that history dependence was also exhibited for graphene oxide in ref. 14, where nitrogen dioxide and hydrogen were tested, using absorption intensity measurements^14^.

### Performance

**Figure 7:**
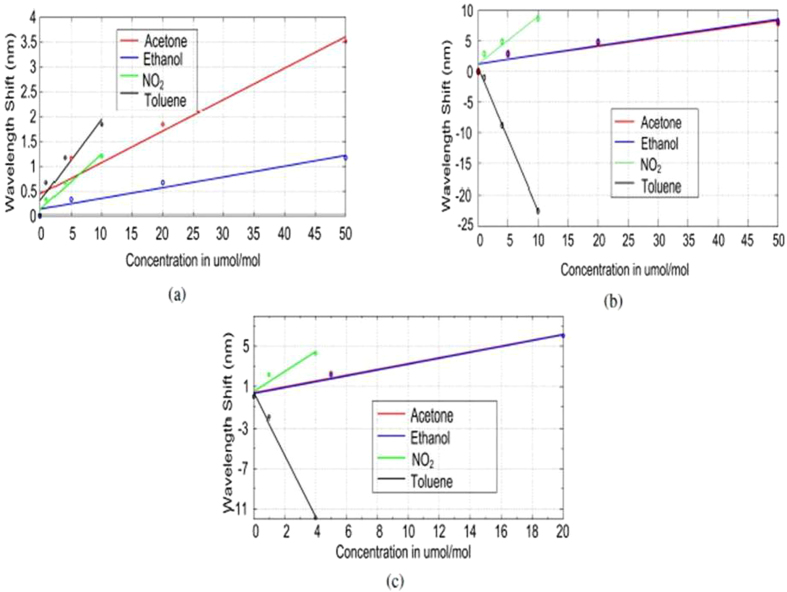
Linear fits for (**a**) bare ONA, (**b**) Graphene oxide covered ONA and (**c**) monolayer graphene covered ONA response to different analytes.

Graphene, though apparently one atom thick when placed on top of the bare ONA, results in a shift of 13 nm, and graphene oxide having multiple layers, results in a shift of 45 nm. Response strengths of the three types of sensors to the four analytes are plotted as a function of analyte concentration in Fig. 7. As we see, the shift in the resonance frequency for both acetone and ethanol is very similar for graphene or graphene oxide covered ONA. The maximum shift is observed for toluene which has highest refractive index amongst all the analytes. The shift for nitrogen dioxide and toluene are also caused by changes occurring in the graphene or graphene oxide layers in addition to the refractive index changes. The sensitivity and the limit of detection is as shown in Table 2. As we see from the table, the sensitivity is given in nm/(*μ*mol/mol) and detection limit is calculated as the smallest concentration that can give a spectral shift detected by the spectrometer. The slope was calculated using a linear fit on each sensitivity curve shown in Fig. 7.

From Table 2 we can say that graphene oxide and graphene covered ONA have a larger responses as compared to bare ONA. The responses of graphene oxide and graphene are very similar. Graphene oxide, having multiple layers, has a generally higher capacity for holding different analyte molecules. For any given analyte, the response of graphene covered ONA saturates at a lower concentration than that of graphene oxide covered ONA. However, until saturated, the responses of graphene oxide covered ONA and graphene covered ONA to different analytes are very similar. This optical behavior is unlike conductance measurement techniques, where the response degrades when graphene oxide is used instead of graphene. As already noted, a characteristic observed in graphene oxide or graphene coated ONA sensors is that the response does not return to its baseline. The overall performance is summarized in the Table 3. Apparent selectivity is offered by the change in the direction of the resonance peak shift either blue or red. For the bare ONA, a red shift is observed for all analytes (Δ*λ* > 0). For ONA covered with graphene or graphene oxide depending on the analyte, the shift is either blue (Δ*λ* < 0) or red (Δ*λ* > 0). However, there may be an adsorbate history contribution to this effect (as discussed below).

### Response Phenomena

The bare ONA resonates at 746.2 nm. As indicated above, when a monolayer of graphene is placed on top of the bare ONA to form graphene covered ONA, the resonance frequency is red shifted to 759 nm. The shift of around 13 nm was expected due to graphene as seen before^12^. When graphene oxide was drop cast on bare ONA, the resonance wavelength exhibited a red shift of around 45 nm.

The baseline values before and after analyte adsorption for the bare ONA, graphene covered ONA and graphene oxide covered ONA are given in Table 4. The baseline value tabulations show that there is a significant baseline drift exhibited in sensing gases for the graphene covered ONA and for the graphene oxide covered ONA. However, the response for the bare ONA comes back to its baseline once the analyte flow is stopped. The baseline changes for analyte exposure of graphene and graphene oxide covered ONA seem to imply that there are residual analyte molecules remaining on graphene or graphene oxide. Note that the response of monolayer graphene covered ONA and multilayer graphene oxide covered ONA are similar. This may be because only the molecules closest to the ONA may be dominantly influencing the response.

**Figure 8:**
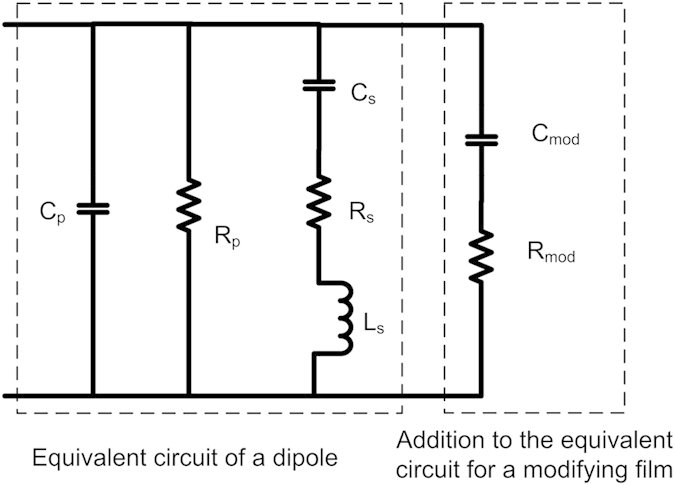
Equivalent circuit for the dipole nano antenna covered with graphene/graphene oxide.

The red shifts observed in the responses of the bare ONA can also be explained with the help of the left portion of the equivalent circuit diagram in Fig. 8^12^. In the equivalent circuit diagram, the two arms of the dipole are represented by L_s_ and R_s_. The gap between the two arms of the dipole is modeled by C_s_. C_p_ and R_p_ denote capacitance and resistance connected to the ambient conditions^12^,^15^,^16^. The analyte molecules form a small layer on top of the dipole structure. The thickness of this layer formed is very small as the molecules are not attached to gold. The analyte molecules coming in the vicinity of the dipole change the value of C_s_, and more dominantly C_p_. Bare ONA has a fast response and is reversible, as the response comes back to its baseline value once the analyte flow is stopped. We also observe that the response saturates at higher concentrations. In the equivalent circuit diagram this corresponds to the larger values reached by capacitor C_s_ and C_p_. The bare ONA response has no obvious selectivity towards any analyte. The bare ONA gives a red shift to all the analytes, making it hard to differentiate between analytes. However, among all the four analytes tested, bare ONA exhibited its maximum sensitivity towards toluene and nitrogen dioxide.

The shift in resonance frequency for graphene or graphene oxide covered ONA is a competition between two phenomena. First, there is the change in the bulk refractive index around the ONA dipole as described by Equation 2. This is represented in Fig. 8 as changes to C_p_ and R_p_, and would yield a red shift in the peak resonance since all the analytes have a refractive index larger than air (Table 1). Second, there is a possibility for reduction/oxidation of the coating layer of the ONA. This is due to the analyte gas, resulting in a change in the optical conductivity of the graphene or graphene oxide. These changes are represented by the changes in the values of C_mod_ and R_mod_ in Fig. 8. We represent graphene/graphene oxide by a capacitor and a resistor because graphene or graphene oxide does not exhibit plasmon resonance in the visible spectrum^12^,^16^. Reducing gases would transfer electrons to the coating layer, yielding a blue shift, while oxidizing gases would remove electrons yielding a red shift. Because red shifts in the peak resonance are observed upon interaction with acetone and ethanol (reducing gases), it suggests that the changes in refractive index are dominating the sensor responses. In the electrical equivalent circuit, the red shift by acetone and ethanol is the result of a dominant effect of the increase in C_p_ on the resonant wavelength over any reduction in C_mod_. For nitrogen dioxide, an oxidizing gas, both the change in refractive index and the change in the chemical potential of the sensor support a red shift of the peak resonance. In the electrical equivalent circuit, it can be said that nitrogen dioxide increases the value of C_p_ and C_mod_ resulting in a red shift in the resonance wavelength.

**Figure 9:**
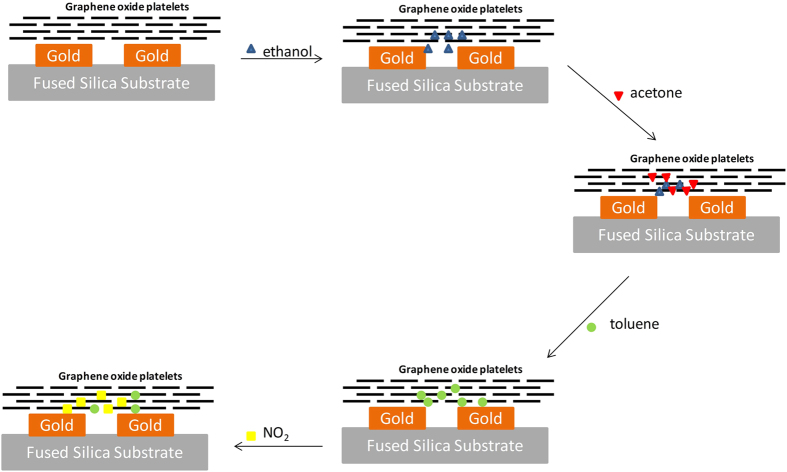
A schematic representation of the step-wise interaction of the analyte molecules with the modified ONA sensors. For simplicity, background molecules (air) are not included, and only a qualitative indication of analyte molecules at the dipole is shown.

The blue shift observed for toluene, a reducing gas, would suggest that the electronic interactions are dominating the sensor response, despite the relatively large refractive index associated with toluene. Toluene, with respect to acetone and ethanol, has been shown to interact with graphene relatively strongly^17^ which may lead to larger changes in the chemical potential of the sensor. Furthermore, as we noted above, there are history effects associated with the ONA sensor modified with graphene and graphene oxide: it appears that analyte molecules are sticking in or on the modifying layer, changing the prior baseline resonance wavelength (Fig. 9). It is possible that as toluene interacts with the modifying layer, it displaces residual acetone and ethanol owing to its stronger interaction. Because it is displacing other organic molecules, the effective change in refractive index is smaller than if the toluene molecules were displacing air (see Equation 2). Because this bulk refractive index change is reduced in magnitude, the reducing nature of the toluene may dominate, leading to the observed blue shift in peak resonant wavelength. In the equivalent electrical circuit, the shift in the resonance wavelength by the increase in C_p_ is dominated by the decrease in the value of C_mod_.

Our suggested mechanism helps to explain the observed shifts seen in our testing sequence. However, we have no independent measurements at this time to verify these suggested effects. In general, the sensor system using the modifying layer is a more complicated system, with history effects and the potential for interactions between co-adsorbates confounding the sensor responses. Despite the complicating factors, these sensor configurations do offer higher sensitivity when compared to the bare system. Further investigations are planned to determine the role of analyte history on sensor performance, and to develop approaches that can remove residual adsorbates. These could include dosing the sensor with UV or thermal pulses, methods which have been previously shown to be effective in cleaning graphene and graphene oxide films^1^,^18^.

## Conclusion

In this work, we studied three different configurations of ONA for gas phase chemical sensing. The bare ONA sensor has a fast and reversible response without any baseline drift, but also has lowest sensitivity among all the three configurations of sensors. Due to the changes in the refractive index by the different analyte, there was always a red shift observed for bare ONA structure. The presence of modifying layers on top of the ONA makes a difference in the response characteristics. Graphene and graphene oxide coated structures demonstrated higher sensitivity as compared to bare ONA. Wavelength shift was dependent on the analytes tested. It was either blue shifted (for toluene) or red shifted (for acetone, ethanol and nitrogen dioxide). A possible mechanism underlying the wavelength shift for graphene and graphene oxide coated ONA is discussed. Baseline drift was observed in the response of the graphene and graphene oxide coated ONA. Graphene and graphene oxide coated ONA have a slower response as compared to bare ONA.

## Additional Information

**How to cite this article**: Mehta, B. *et al.* Gas Sensing with Bare and Graphene-covered Optical Nano-Antenna Structures. *Sci. Rep.* **6**, 21287; doi: 10.1038/srep21287 (2016).

## Supplementary Material

Supplementary Information

## Figures and Tables

**Table 1:** Refractive indices of different analytes in liquid phase (or coverings on top of ONA) at 600 nm.

Covering/Analytes	Refractive index (n, k)
Graphene/graphene oxide	(2.58, 1.38)^19^
Acetone	(1.3586, 0)
Ethanol	(1.3612, 0)
Toluene	(1.4958, 0)
Nitrogen dioxide	(1.449, 0)

**Table 2:** Sensitivity of different sensors for different analytes.

Device	Acetone		Ethanol		Nitrogen Dioxide		Toluene	
	Sensitivity (nm/*μ*mol/mol)	Detection Limit (*μ*mol/mol)	Sensitivity (nm/*μ*mol/mol)	Detection Limit (*μ*mol/mol)	Sensitivity (nm/*μ*mol/mol)	Detection Limit (*μ*mol/mol)	Sensitivity (nm/*μ*mol/mol)	Detection Limit (*μ*mol/mol)
Bare ONA	0.06	2.29	0.022	6.70	0.11	1.30	0.16	0.89
GO covered ONA	0.15	1.03	0.15	1.00	0.78	0.19	2.31	0.06
MLG covered ONA	0.29	0.50	0.29	0.50	0.99	0.15	3.05	0.05

**Table 3:** Performance analysis of different configurations of ONA.

Device	Sensitivity	Response time	Reversibility
Bare ONA	Least sensitive	Fastest	Reversible
Graphene oxide covered ONA	Highly sensitive	Relatively slow response	Baseline drift
Monolayer graphene covered ONA	Highly sensitive	Relatively slow response	Baseline drift

**Table 4:** Baseline values of resonance frequency at the start and end of all the analytes on bare, graphene covered, GO covered ONA.

Analyte	Bare ONA		Graphene covered ONA		GO covered ONA	
	Start	End	Start	End	Start	End
Acetone	746.2	746.2 (a)	759.0	769.0 (a)	797.5	799.5 (b)
Ethanol	746.2	746.2 (b)	769.0	776.7 (b)	790.5	797.5 (a)
Toluene	746.2	746.2 (c)	776.7	769.1 (c)	799.5	790.5 (c)
NO_2_	746.2	746.2 (d)	769.1	776.8 (d)	790.5	797.5 (d)

a, b, c and d give exposure order for each of the different ONAs. See Fig. 3d for the starting resonance wavelength positions for each of the three sensor types.
