# Supplementary material for: Gas Sensing with Bare and Graphene-covered Optical Nano-Antenna Structures
Source: Sci Rep. 2016 Feb 17;6:21287. doi: 10.1038/srep21287 (PMC4756381; doi:10.1038/srep21287)
Supplement: Supplementary Information [file srep21287-s1.pdf]

Gas Sensing with Bare and Graphene-covered Optical Nano-Antenna Structures

Bhaven Mehta[1], Kurt D. Benkstein[2], Steve Semancik[2], Mona E. Zaghloul[1]
[1]Department of Electrical and Computer Engineering,
The George Washington University, Washington DC, 20037
[2]Biomolecular Measurement Division, National Institute of
Standards and Technology, Gaithersburg, MD 20899-8362
bhavenm@gwmail.gwu.edu, kurt.benkstein@nist.gov,
stephen.semancik@nist.gov, zaghloul@gwu.edu

December 1, 2015

Supporting Information

1 Comparison between the experimental and simulated data

The FDTD simulations were done using Lumerical's Finite Difference Time Domain (FDTD) software (www.lumerical.com) and the measured outputs are as shown in Figure S1. There is a difference in the measured results and the simulated results as shown in Figure S1. This is because of variations introduced in the fabrication process. The fabricated structures do not have exact rectangles with sharp corners but they have rounded corners as shown in Figure 2b in the manuscript. Due to rounding of the corners, the new structures have less volume than the desired structures. This results in a blue shift in the resonance frequency [1, 2]. A comparison between the experimental measured peaks and the simulation of the rounded structures is shown in the Figure S2.

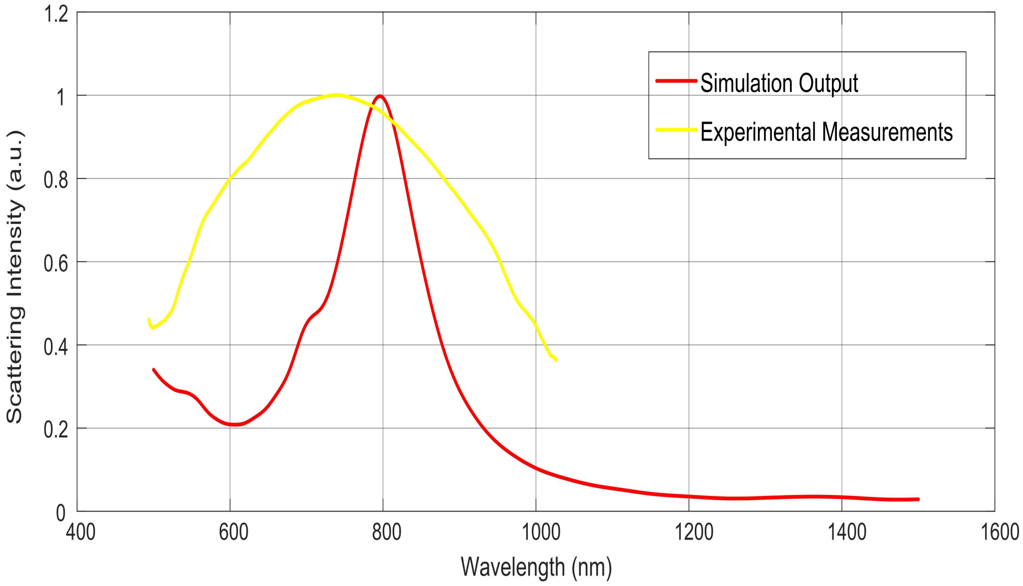

Figure S1: Comparison between the simulation and experimental results

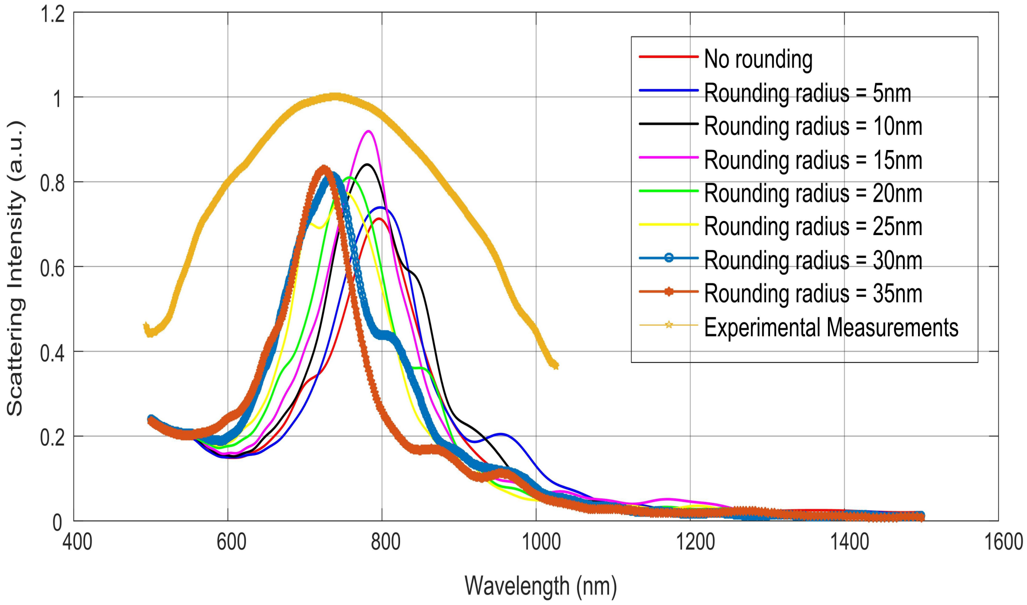

Figure S2: Effect of rounding on the resonance wavelength of the dipole structures.

We also observe that the measured response is broad. This can be explained as follows.

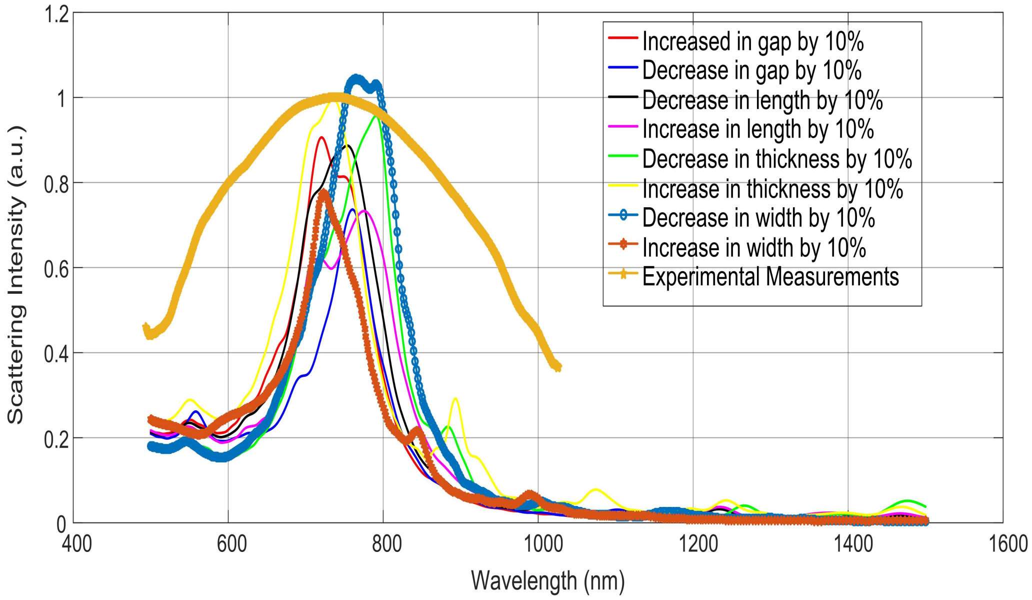

Figure S3: Effect of the changes in the dimensions of the dipole structure due to random variations in the fabrication process

The fabricated sample on an area of 1mm $\times$ 1mm is composed of more than 250,000 dipole structures ONA. There are more than 250,000 ONA in the given sample. Fabrication process involved a error of 10%. Due to this error there are random variations introduced in the dimensions of the ONA. This results in a shift in the resonance frequency of the ONA from the designed ONA. When the source input is applied on the 1mmx1mm substrate all of the 250,000, all of the ONA's are active at the same time. The result is the sum of all of the spectrum of the active nano structures, and it will cause the broadening of the bandwidth of the measured response as compared to the simulated response of the ONA. This results in a ONA having different resonant frequencies which are closely placed. This is shown in Figure S3. When the spectrum is measured, all the 200,000+ ONA antennas are measured simultaneously. So this results in a broadening of the curve compared to the theoretically expected curve.

2 Raman Spectrum

The Raman Spectrum for graphene covered on ONA and Graphene oxide covered ONA is as shown in Figure S4 and in Figure S5.

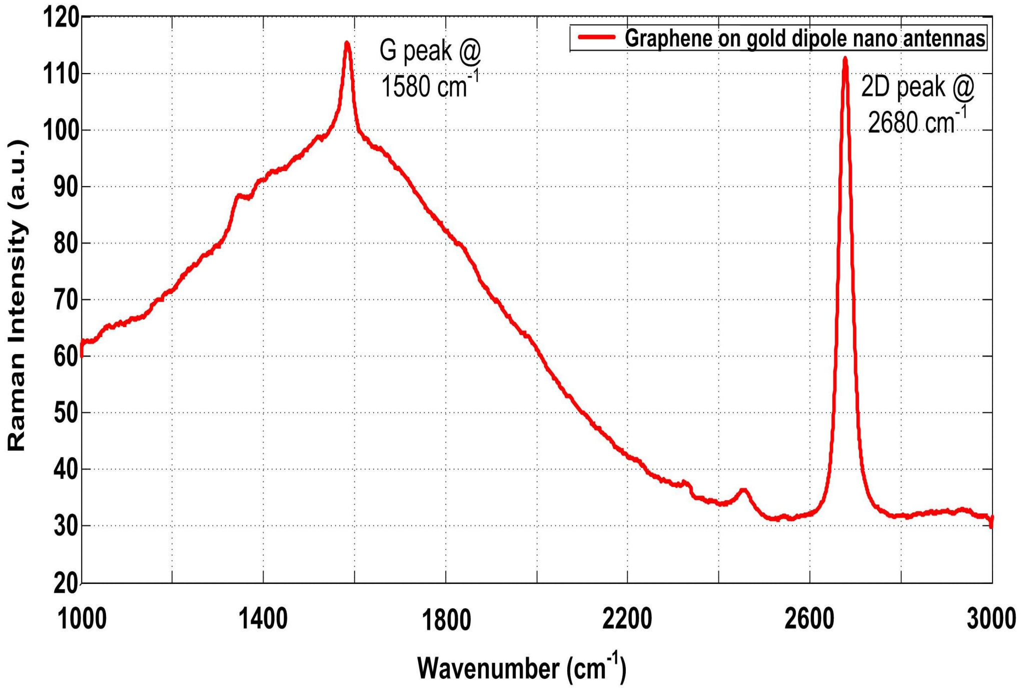

Figure S4: Raman Spectrum of monolayer graphene on gold dipole nano antennas

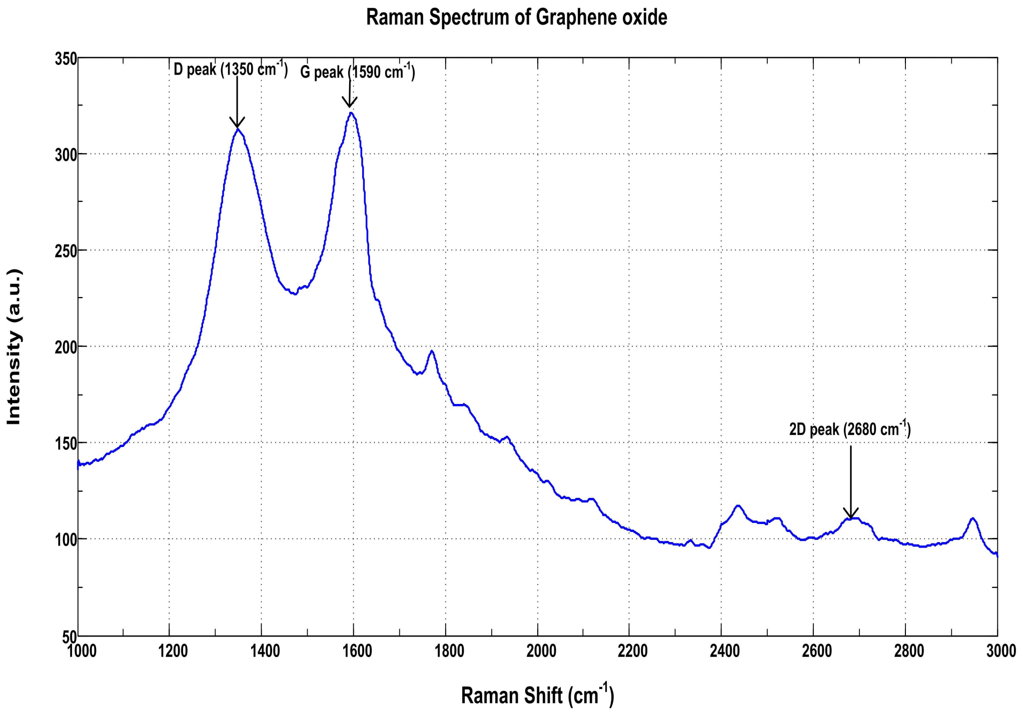

Figure S5: Raman Spectrum of graphene oxide on gold dipole nano antennas

The Raman Spectrum was measured using Horiba's LabRAM HR Raman Spectroscope. 532.8nm laser was used. Exposure time of 5 seconds and accumulation number of 5 was set. Number of layers of graphene is determined ratio of the intensity of the 2D peak (at 2680 cm^{-1}) to the intensity of G peak (at 1580 cm^{-1}) (I_{2D}/I_G). For monolayer graphene this ratio is more than 1 (around 4) [3]. For multiple layers of graphene the ratio is decreasing. For four or more layers, the ratio is less than 1.

For graphene oxide, as shown in Figure S5 there is a prominent D peak (at 1350 cm^{-1}) as expected. The D peak at 1350 cm^{-1} implies that there are defects in graphene such as the edges, which are present in graphene oxide.

References

[1] F Pelayo Garc X Ed a De Arquer, Vladimir Volski, Niels Verellen, Guy a E Vandenbosch, and Victor V Moshchalkov. Engineering the Input Impedance of Optical Nano Dipole Antennas: Materials, Geometry and Excitation Effect. *IEEE Transactions on Antennas and Propagation*, 59(9):3144–3153, 2011. URL http://ieeexplore.ieee.org/lpdocs/epic03/wrapper.htm?arnumber=5948358.

[2] B. Mehta and M. E. Zaghloul. Tuning the Scattering Response of the Optical Nano Antennas Using Graphene. *IEEE Photonics Journal*, 6(1):1–8, February 2014. ISSN 1943-0655. doi: 10.1109/JPHOT.2014.2300501. URL http://ieeexplore.ieee.org/lpdocs/epic03/wrapper.htm?arnumber=6716956.

[3] Xuesong Li, Weiwei Cai, Jinho An, Seyoung Kim, Junghyo Nah, Dongxing Yang, Richard Piner, Aruna Velamakanni, Inhwa Jung, Emanuel Tutuc, et al. Large-area synthesis of high-quality and uniform graphene films on copper foils. *Science*, 324(5932):1312–1314, 2009.
